# The absence of the *drhm* gene is not a marker for human-pathogenicity in European *Anaplasma phagocytophilum* strains

**DOI:** 10.1186/s13071-020-04116-z

**Published:** 2020-05-07

**Authors:** Denis B. Langenwalder, Sabine Schmidt, Cornelia Silaghi, Jasmin Skuballa, Nikola Pantchev, Ioana A. Matei, Andrei D. Mihalca, Urs Gilli, Joanna Zajkowska, Martin Ganter, Tove Hoffman, Erik Salaneck, Miroslav Petrovec, Friederike D. von Loewenich

**Affiliations:** 1 Department of Medical Microbiology and Hygiene, Medical Center of the Johannes Gutenberg-University Mainz, Obere Zahlbacherstrasse 67, 55131 Mainz, Germany; 2 Institute of Infectology, Friedrich-Loeffler-Institut, Südufer 10, 17493 Greifswald-Insel Riems, Germany; 3 Chemical and Veterinary Investigations Office Karlsruhe (CVUA Karlsruhe), Weissenburgerstrasse 3, 76187 Karlsruhe, Germany; 4 IDEXX Laboratories, Mörikestrasse 28/3, 71636 Ludwigsburg, Germany; 5 Department of Parasitology and Parasitic Diseases, University of Agricultural Sciences and Veterinary Medicine of Cluj-Napoca, Calea Manastur 3-5, 400372 Cluj-Napoca, Romania; 6 IDEXX Diavet AG, Schlyffistrasse 10, 8806 Bäch, Switzerland; 7 Department of Infectious Diseases and Neuroinfections, Medical University of Białystok, ul.Żurawia 14, 15-345 Białystok, Poland; 8 Clinic for Swine and Small Ruminants, University of Veterinary Medicine Hannover, Bischofsholer Damm 15, 30173 Hannover, Germany; 9 Department of Medical Biochemistry and Microbiology (IMBIM), Zoonosis Science Center, Uppsala University, Uppsala, Sweden; 10 Department of Medical Sciences, Zoonosis Science Center, Uppsala University, Uppsala, Sweden; 11 Institute of Microbiology and Immunology, Faculty of Medicine, University of Ljubljana, Zaloška 4, 1000 Ljubljana, Slovenia

**Keywords:** *Anaplasma phagocytophilum*, *ankA*, APH_0919, APH_0922, Asia, *drhm*, Europe, Human, Multilocus sequence typing (MLST), North America, Pathogenicity

## Abstract

**Background:**

*Anaplasma phagocytophilum* is a Gram-negative obligate intracellular bacterium that replicates in neutrophil granulocytes. It is transmitted by ticks of the *Ixodes ricinus* complex and causes febrile illness in humans and animals. The geographical distribution of *A. phagocytophilum* spans the Americas, Europe, Africa and Asia. However, human disease predominantly occurs in North America but is infrequently reported from Europe and Asia. In North American strains, the absence of the *drhm* gene has been proposed as marker for pathogenicity in humans whereas no information on the presence or absence of the *drhm* gene was available for *A. phagocytophilum* strains circulating in Europe. Therefore, we tested 511 European and 21 North American strains for the presence of *drhm* and compared the results to two other typing methods: multilocus sequence typing (MLST) and *ankA*-based typing.

**Results:**

Altogether, 99% (478/484) of the analyzable European and 19% (4/21) of the North American samples from different hosts were *drhm-*positive. Regarding the strains from human granulocytic anaplasmosis cases, 100% (35/35) of European origin were *drhm-*positive and 100% (14/14) of North American origin were *drhm-*negative. Human strains from North America and Europe were both part of MLST cluster 1. North American strains from humans belonged to *ankA* gene clusters 11 and 12 whereas European strains from humans were found in *ankA* gene cluster 1. However, the North American *ankA* gene clusters 11 and 12 were highly identical at the nucleotide level to the European cluster 1 with 97.4% and 95.2% of identity, respectively.

**Conclusions:**

The absence of the *drhm* gene in *A. phagocytophilum* does not seem to be associated with pathogenicity for humans *per se*, because all 35 European strains of human origin were *drhm-*positive. The epidemiological differences between North America and Europe concerning the incidence of human *A. phagocytophilum* infection are not explained by strain divergence based on MLST and *ankA* gene-based typing.
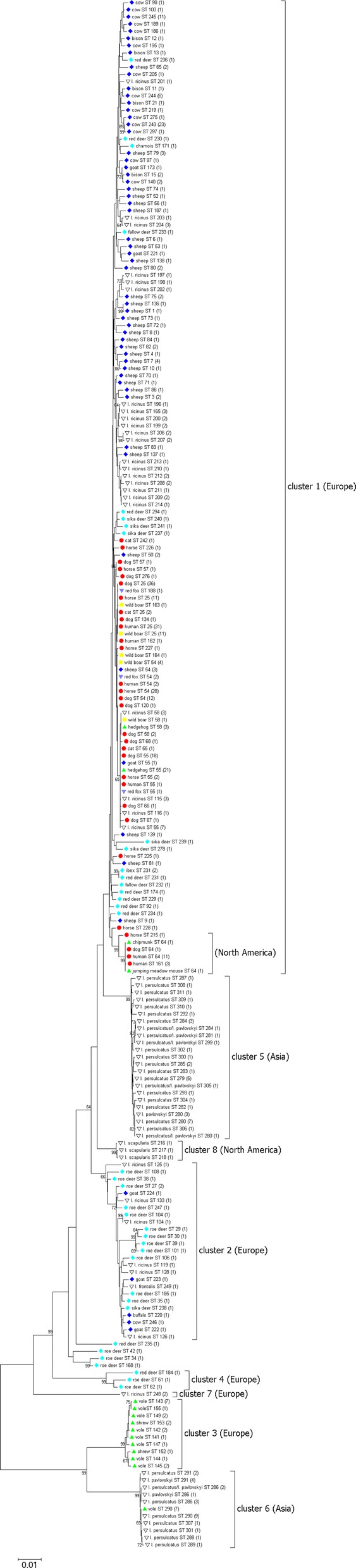

## Background

*Anaplasma phagocytophilum* is a Gram-negative obligate intracellular bacterium that replicates in neutrophil granulocytes [1]. It causes febrile illness in humans and animals and is transmitted by ticks of the *Ixodes ricinus* complex [2, 3]. The main vectors of *A. phagocytophilum* are *I. ricinus* in much of Europe, *I. persulcatus* in north-eastern Europe and East Asia and *I. scapularis* and *I. pacificus* in North America [2]. *Anaplasma phagocytophilum* has a wide geographical distribution that spans the Americas, Europe, Africa and Asia [2]. However, human disease predominantly occurs in North America with 4008 anaplasmosis cases in 2018 in the USA (https://wonder.cdc.gov/nndss/nndss_annual_tables_menu.asp?mmwr_year=2018). In contrast, human granulocytic anaplasmosis is infrequently reported from Europe [4] and Asia [5–9]. Of note, most patients from China initially described as to be infected by *A. phagocytophilum* suffered from a bunyavirus infection called severe fever with thrombocytopenia syndrome (SFTS) [5, 10–12].

*Anaplasma phagocytophilum* is not only geographically widely distributed, but also has a broad host range. Clinically apparent disease is mainly observed in humans [3], dogs [13], horses [14], cats [15] as well as in sheep and cattle [16]. Symptomatic granulocytic anaplasmosis in domestic ruminants has been observed in Europe [17], Africa [18, 19], the Near [20] and Far East [21], whereas it has not been reported from North America so far [22].

Transovarial transmission of *A. phagocytophilum* is inefficient at least in *Ixodes* ticks [23, 24]. It is therefore thought to depend on reservoir hosts to complete its life-cycle. The white-footed mouse (*Peromyscus leucopus*) is probably the main reservoir for human infection in the USA [25, 26], whereas the situation is less clear for Europe. Several species including wild ruminants, small mammals and wild boar have been considered in the past [22].

A variety of single and multilocus sequence typing schemes have been used (i) to elucidate the epidemiological differences mentioned above; (ii) to find markers for human pathogenicity; (iii) to study host adaptation of distinct *A. phagocytophilum* strains; and (iv) to determine reservoir hosts for human and animal infection [22]. The absence of the *drhm* gene has been proposed as a marker for pathogenicity in humans and dogs when seven whole genome sequences from five North American and two European strains from different hosts were compared [27]. However, this could not be verified on a larger series of 117 samples from the USA because 25% (4/16) of dog strains were *drhm* positive [28]. No information on the presence or absence of the *drhm* gene has been available so far for *A. phagocytophilum* circulating in Europe.

Therefore, we here tested 511 European and 21 North American *A. phagocytophilum* strains for the presence of *drhm* and compared the results to two other typing methods that we found in the past to have high discriminatory power: multilocus sequence typing (MLST) and *ankA*-based typing [29, 30].

## Methods

### Samples

In total, 686 *A. phagocytophilum* strains were included. Of them, 98 were from this study and originated from 3 humans, 35 domestic animals (18 horses, 14 dogs, 2 cats and 1 cow), 57 wild animals (19 red deer, 9 roe deer, 9 sika deer, 6 wild boars, 5 mouflons, 4 fallow deer, 2 ibexes, 2 red foxes and 1 bird) and 3 ticks (2 *I. ricinus* and 1 *I. frontalis*). The two nymphs and one adult female were engorged and removed from blackbirds (*Turdus merula*). A total of 577 strains were reported previously. All human strains originated from patients with human granulocytic anaplasmosis. Reference, host species, country of origin, year of sampling and disease state of the host are shown in Additional file 1: Table S1.

### Presence or absence of the *drhm* gene

The *drhm* sequences available at GenBank were aligned by ClustalW. Four primers, drhm 1f (5′-CGT CAT GTG CAC TAA TAG CC-3′), drhm 2r (5′-CTC TCA TGA AAA CTA GAC GAT-3′), drhm 3f (5′-GCT ATT GCA ACA GTA ATG ACT-3′) and drhm 4r (5′-GGT ATG GTT CCA TTC TCC TG-3′) were designed for nested amplification of a 392-bp fragment. Primers drhm 1f and drhm 2r were used in the first and drhm 3f and drhm 4r in the second PCR round.

The genomic region, which contains the *drhm* gene, was partially amplified as well to prove its presence. It is flanked by the inversely duplicated genes APH_0919 and APH_0922 [27]. APH_0919 and APH_0922 sequences were extracted from the *A. phagocytophilum* whole genome sequences available on GenBank and aligned by ClustalW. Degenerated primers APH_0919 1f (5′-ATA TCC CTG CCR TTT RTK CTG), APH_0919 2r (5′-GTG GTA YAT TAG ATG TAT CAA AA-3′), APH_0919 3f (5′-CTG CTT CAY GCA ACS CTR TTA-3′) and AHP_0919 4r (5′-TTA GAT GTA TCA AAA CAY ATT GC-3′) were designed to amplify a 482-bp fragment of APH_0919 and/or APH_0922. Primers APH_0919 1f and APH_0919 2r were applied in the first and APH_0919 3f and APH_0919 4r in the second PCR round.

Two μl of DNA were used as a template in a 50 μl reaction mixture containing 50 mM KCl, 20 mM Tris-HCl (pH 8.4), 2 mM MgCl_2_, 0.2 mM deoxynucleoside triphosphates, 0.4 μM concentrations of each primer and 0.2 μl (1 U) of *Taq* DNA polymerase (Invitrogen, Karlsruhe, Germany). The PCR were performed by a 2720 GeneAmp thermal cycler (Applied Biosystems, Darmstadt, Germany) under the following conditions: an initial denaturation at 94 °C for 3 min; followed by 40 cycles of denaturation at 94 °C for 30 s, annealing at 54 °C for 30 s, extension at 72 °C for 30 s; and a final extension step at 72 °C for 10 min. Individual *drhm* and APH_0919/APH_0922 amplicons were bidirectionally sequenced to prove PCR specificity.

### MLST and *ankA* gene

Seven housekeeping genes (*pheS*, *glyA*, *fumC*, *mdh*, *sucA*, *dnaN* and *atpA*) were amplified for MLST and sequenced bidirectionally as reported previously [29]. Clonal complexes (CC) were defined by sharing identical alleles at five of the seven loci with at least one other member of the group. The *ankA* gene was partially amplified and bidirectionally sequenced as described [29]. Full length *ankA* sequences were obtained in tick_CM20 and tick_CS2 (cluster 6) as detailed in Additional file 2: Text S1, and in horse_S1523_07 (cluster 7) as described previously [31].

### Phylogenetic analysis

Sequences were codon-aligned by ClustalW applying the PAM (Dayhoff) matrix. Trees were constructed using the neighbor-joining (NJ) method with the Jukes-Cantor model and the complete deletion option in the program MEGA X version 10.0.5 [32]. Bootstrap analysis was conducted with 1000 replicates. Net average distances between nucleotide sequences of MLST and *ankA* gene clusters were computed using the Jukes-Cantor matrix and applying the complete deletion option. Net average distances between protein sequences of *ankA* gene clusters were calculated using the PAM (Dayhoff) matrix and applying the complete deletion option.

### Comparison of typing methods

To test for the concordance between different typing methods, adjusted Wallace coefficients [33] were calculated using the online tool available at: http://www.comparingpartitions.info/index.php?link=Tool. For example, the Wallace coefficient host → MLST cluster is the probability that two strains are found in the same MLST cluster, if they are from the same host.

## Results

### Presence or absence of the *drhm* gene

The presence or absence of the *drhm* gene was determined in 532 *A. phagocytophilum* strains, 511 from Europe and 21 from North America (Additional file 1: Table S1). The information was extracted from GenBank in 13 cases (3 from Europe, 10 from the USA). The DNA was used up or information on *drhm* was not available on GenBank in 154 of the 668 strains in total.

The absence of a gene is difficult to prove due to methodical reasons. Therefore, the amplification of the flanking genes APH_0919/APH_0922 was undertaken in 519 samples in order to prove that the genomic region containing the *drhm* gene was present (Additional file 1: Table S1). This information was extracted from GenBank in 13 cases. 95% (505/532) of the *A. phagocytophilum* strains investigated contained the APH_0919 and/or APH_0922 gene (Table 1). The 27 APH_0919/APH_0922-negative samples were exclusively of European origin and from 22 voles, 3 shrews and 2 *I. ricinus* ticks removed from blackbirds (*T. merula*) (Table 2). All APH_0919/APH_0922-negative samples were also negative for *drhm* (Table 1).

**Table 1:** Number and origin of the APH_0919/APH_0922 and *drhm* gene positive and negative *A. phagocytophilum* strains

	*drhm*-positive		*drhm*-negative		Total
APH_0919/APH_0922-positive	478	Europe	6	Europe	484
	4	North America	17	North America	21
APH_0919/APH_0922-negative	0		27	Europe	27
Total	482		50		532

**Table 2:** Number, origin and host of the APH_0919/APH_0922 and *drhm* gene positive and negative *A. phagocytophilum* strains

Continent	Host species
APH_0919/APH_0922-positive, *drhm*-positive (*n* = 482)	
Europe (*n* = 478)	Humans (*Homo sapiens*) (*n* = 35)
	Domestic animals (*n* = 235)
	Dogs (*Canis lupus familiaris*) (*n* = 67)
	Horses (*Equus caballus*) (*n* = 44)
	Cats (*Felis catus*) (*n* = 3)
	Cattle (*Bos taurus*) (*n* = 62)
	Sheep (*Ovis aries*) (*n* = 52)
	Goats (*Capra aegagrus hircus*) (*n* = 6)
	Water buffalo (*Bubalus bubalis*) (*n* = 1)
	Large wild animals (*n* = 144)
	Roe deer (*Capreolus capreolus*) (*n* = 51)
	Red deer (*Cervus elaphus*) (*n* = 33)
	Sika deer (*Cervus nippon*) (*n* = 9)
	Fallow deer (*Dama dama dama*) (*n* = 4)
	European bison (*Bison bonasus*) (*n* = 15)
	Mouflon (*Ovis gmelini musimon*) (*n* = 5)
	Chamois (*Rupicapra rupicapra*) (*n* = 3)
	Ibex (*Capra ibex*) (*n* = 2)
	Wild boar (*Sus scrofa*) (*n* = 18)
	Red foxes (*Vulpes vulpes*) (*n* = 4)
	Small mammals (*n* = 30)
	Hedgehogs (*Erinaceus europaeus*) (*n* = 30)
	Ticks (*n* = 34)
	*I. ricinus* (*n* = 33)
	*I. frontalis* (*n* = 1)
North America (*n* = 4)	Domestic animal (*n* = 1)
	Horse (*Equus caballus*) (*n* = 1)
	Ticks (*n* = 3)
	*I. scapularis* (*n* = 3)
APH_0919/APH_0922-positive, *drhm*-negative (*n* = 23)	
Europe (*n* = 6)	Domestic animals (*n* = 3)
	Dogs (*Canis lupus familiaris*) (*n* = 3)
	Large wild animals (*n* = 3)
	Roe deer (*Capreolus capreolus*) (*n* = 2)
	Red deer (*Cervus elaphus*) (*n* = 1)
North America (*n* = 17)	Humans (*Homo sapiens*)^a^ (*n* = 14)
	Domestic animal (*n* = 1)
	Dog (*Canis lupus familiaris*) (*n* = 1)
	Small mammals (*n* = 2)
	Jumping meadow mouse (*Zapus hudsonius*) (*n* = 1)
	Chipmunk (*Tamias striatus*) (*n* = 1)
APH_0919/APH_0922-negative, *drhm*-negative (*n* = 27)	
Europe (*n* = 27)	Small mammals (*n* = 25)
	Voles (*Myodes glareolus*, *n* = 19; *Microtus arvalis*, *n* = 3)
	Shrews (*Sorex araneus*) (*n* = 3)
	Ticks (*n* = 2)
	*I. ricinus* (*n* = 2)

^a^Two strains have been reported previously [27]

Of the APH_0919/APH_0922-positive samples, 95% (482/505) were also *drhm-*positive. This was true for 99% (478/484) of the European and 19% (4/21) of the North American *A. phagocytophilum* strains (Table 1). The *drhm-*positive samples from the USA originated from 1 horse and 3 *I. scapularis* ticks (Table 2). Six European strains (from 3 dogs, 2 roe deer and 1 red deer) were APH_0919/APH_0922-positive, but *drhm-*negative (Table 2). The 17 APH_0919/APH_0922-positive, but *drhm-*negative samples from the USA originated from 14 humans, 1 dog, 1 jumping meadow mouse (*Zapus hudsonicus*) and 1 chipmunk (*Tamias striatus*).

Regarding the human strains, 100% (35/35) of the strains with European origin were *drhm-*positive and 100% (14/14) of those with North American origin were *drhm-*negative.

### MLST

In general, different sequences of a given locus (*pheS*, *glyA*, *fumC*, *mdh*, *sucA*, *dnaN* and *atpA*) were ascribed a unique, but arbitrary allele number and each unique combination of alleles was assigned a sequence type (ST). Full profiles were obtained for 653 *A. phagocytophilum* strains. Of these, 93 were from this study, 491 were reported previously by our group and in 69 cases the information was extracted from GenBank or PubMLST (Additional file 1: Table S1). Housekeeping gene sequences with double peaks in the chromatograms were regarded as non-typeable. Therefore, a ST could not be ascribed in 139 *A. phagocytophilum* strains revealing a typeability of 79% (514/653). Clonal complexes (CC) were defined by sharing identical alleles at five of the seven loci with at least one other member of the group. CC 18 and CC 19 are newly described and contained two roe deer samples each. Allele numbers, ST, CC and MLST cluster for each *A. phagocytophilum* strain are shown in Additional file 1: Table S1.

**Fig. 1:**
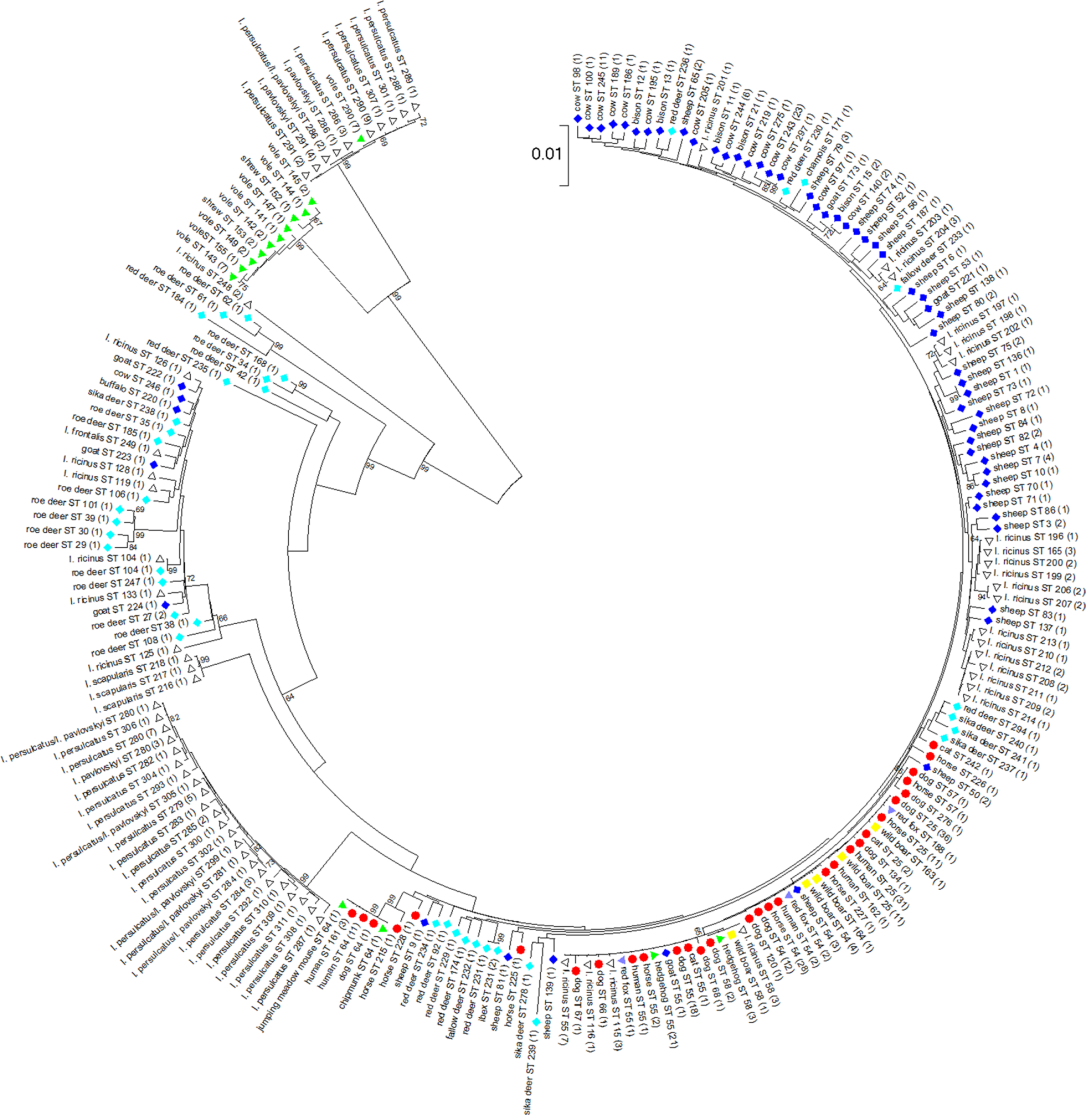
Phylogenetic tree calculated from the concatenated housekeeping gene sequences of 520 samples without ambiguous nucleotides. Tree construction was achieved by the NJ method using the Jukes-Cantor matrix with the complete deletion option. Bootstrap values ≥ 64% are shown next to the branches. The scale-bar indicates the number of nucleotide substitutions per site. The final data set contained 2877 positions. Identical ST are displayed only once per species. The number in parenthesis indicates the frequency with which the respective ST was found. *Key*: red circles, sequences from humans, dogs, horses and cats; dark blue diamonds, sequences from domestic ruminants (cattle, sheep, goats and water buffalo); light blue diamonds, sequences from wild ruminants (roe deer, red deer, sika deer, fallow deer, European bison, mouflon, chamois and ibex); green triangles, sequences from small mammals (hedgehogs, voles, shrews, chipmunk and jumping meadow mouse); yellow squares, sequences from wild boars; purple triangles, sequences from red foxes; white triangles, sequences from ticks

A total of 520 *A. phagocytophilum* strains without ambiguous nucleotides were included in the phylogenetic analysis. The sequences segregated into 8 clusters (Fig. 1, Additional file 3: Fig. S1). Clusters 1 to 3 [29] and 4 to 6 [34, 35] have been described before. Cluster 1 contained strains from humans, domestic animals (dogs, horses and cats), farm animals (cattle, sheep and goats), wild animals (red deer, sika deer, fallow deer, European bison, mouflon, chamois, ibex, wild boar and red foxes), small mammals (hedgehogs, jumping meadow mouse and chipmunk) and *I. ricinus* ticks. Cluster 2 harbored mainly samples from roe deer and *I. ricinus* ticks, but also sporadically sequences from domestic ruminants. Cluster 3 was restricted to strains from voles and shrews from Europe. Cluster 4 was small and constituted by samples from 2 roe deer and 1 red deer. Cluster 5 and cluster 6 contained strains from *I*. *persulcatus*, *I. pavlovskyi* and their hybrids from the Asian part of Russia as described previously [34, 35]. Samples from Asian voles were found exclusively in cluster 6. Cluster 7 harbored 2 strains from *I. ricinus* ticks removed from blackbirds (*Turdus merula*). Cluster 8 contained 3 isolates from *I. scapularis* ticks from the USA that have been classified as non-human pathogenic *A. phagocytophilum* Ap-variant 1 [36]. However, the other North American samples from 11 humans, 1 dog, 1 horse, 1 chipmunk and 1 jumping meadow mouse were part of cluster 1 (Fig. 1, Additional file 3: Fig. S1).

The largest cluster was cluster 1. It contained samples from Europe and North America. However, all other clusters were restricted to samples from either Europe or North America or Asia (Fig. 1, Additional file 3: Fig. S1). Net average distances between the MLST clusters are shown in Table 3. The highest identity of 98.6% was observed between clusters 1 and 8 and the lowest identity of 88.3% between clusters 6 and 8.

**Table 3:** Net average genetic distances between nucleotide sequences of MLST clusters

	Cluster 1	Cluster 2	Cluster 3	Cluster 4	Cluster 5	Cluster 6	Cluster 7
Cluster 2	98.2						
Cluster 3	89.7	89.6					
Cluster 4	94.8	96.4	89.8				
Cluster 5	98.4	97.6	89.6	94.7			
Cluster 6	89.1	88.9	96.0	89.1	89.0		
Cluster 7	92.9	92.9	89.0	92.8	92.4	89.0	
Cluster 8	98.6	98.2	88.9	94.5	97.4	88.3	92.5

### *ankA*

Partial *ankA* sequences were available for 637 *A. phagocytophilum* strains. Of these, 94 were from this study, 491 were reported previously by us and in 52 cases the information was extracted from GenBank (Additional file 1: Table S1). A total of 17 samples from cattle, red deer, roe deer, sika deer and *I. ricinus* were regarded as non-typeable, because they contained *ankA* variants belonging to different clusters (Additional file 1: Table S1). In 15 cases two *ankA* gene variants and in two cases three *ankA* gene variants were present. The typeability regarding the *ankA* gene cluster was 97% (620/637). The *ankA* gene cluster for each *A. phagocytophilum* strain is shown in Additional file 1: Table S1.

**Fig. 2:**
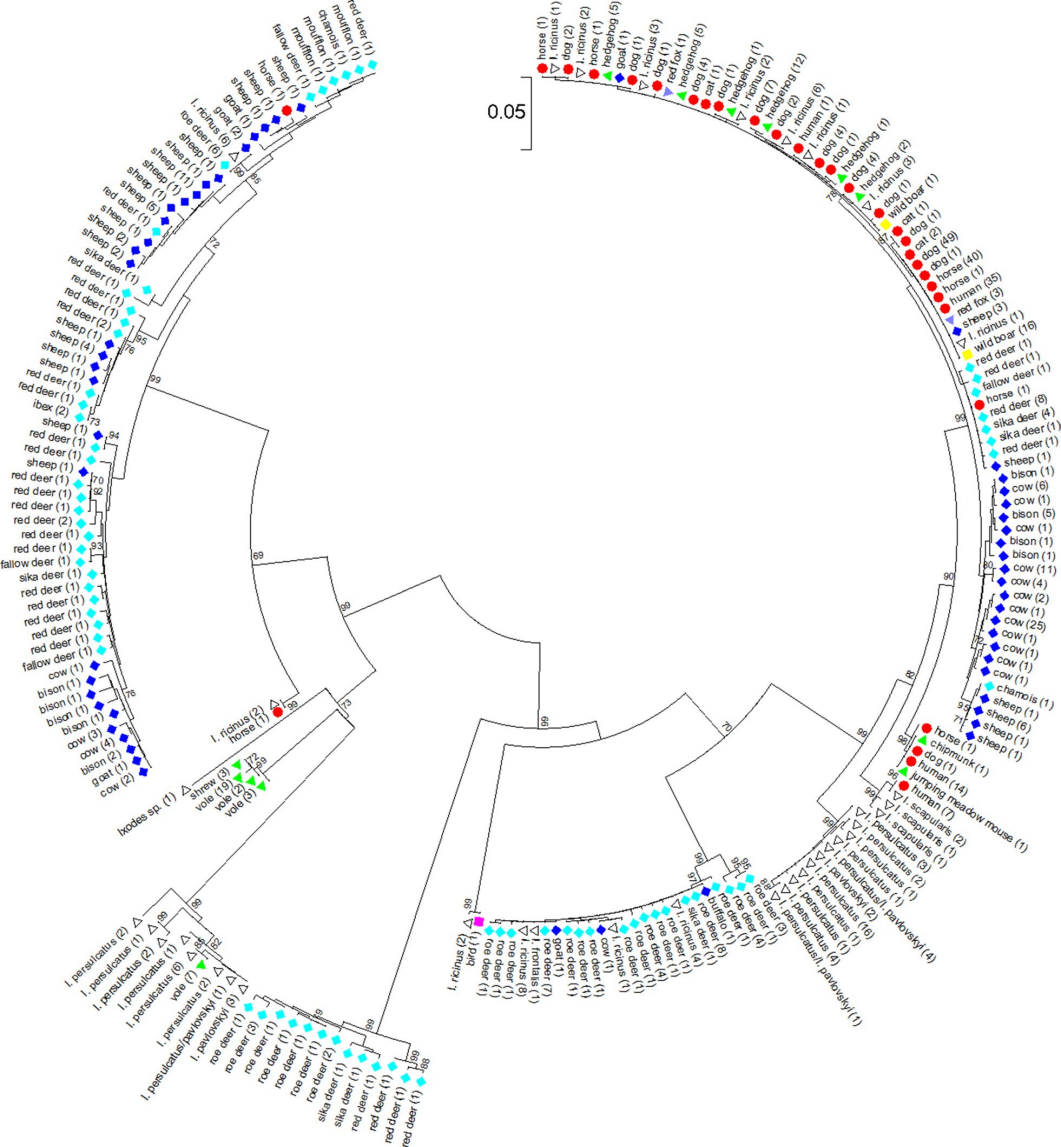
Phylogenetic tree calculated from the *ankA* sequences of 623 samples without ambiguous nucleotides. Tree construction was achieved by the NJ method using the Jukes-Cantor matrix with the complete deletion option. Bootstrap values ≥ 69% are shown next to the branches. The scale-bar indicates the number of nucleotide substitutions per site. The final data set contained 510 positions. Identical *ankA* sequences are displayed only once per species. The number in parenthesis indicates the frequency with which the respective sequence was found. *Key*: red circles, sequences from humans, dogs, horses and cats; dark blue diamonds, sequences from domestic ruminants (cattle, sheep, goats and water buffalo); light blue diamonds, sequences from wild ruminants (roe deer, red deer, sika deer, fallow deer, European bison, mouflon, chamois and ibex); green triangles, sequences from small mammals (hedgehogs, voles, shrews, chipmunk and jumping meadow mouse); yellow squares, sequences from wild boars; purple triangles, sequences from red foxes; pink square, sequence from a bird, white triangles, sequences from ticks

A total of 623 *ankA* sequences without ambiguous nucleotides were included in the phylogenetic analysis. The sequences segregated into 12 clusters (Fig. 2, Additional file 4: Fig. S2). Clusters 1 to 5 [29] as well as 8 and 10 [34] have been described before. Cluster 1 contained strains from humans, domestic animals (dogs, horses and cats), farm animals (cattle, sheep and goats), wild animals (red deer, sika deer, fallow deer, European bison, chamois, wild boar and red foxes), small mammals (hedgehogs) and *I. ricinus* ticks. Cluster 2 harbored mainly samples from roe deer and *I. ricinus* ticks, but also sporadically sequences from domestic ruminants. Cluster 3 comprised mostly strains from roe deer, but also single sequences from red deer and sika deer. Cluster 4 contained samples from one horse, farm animals (cattle, sheep and goats), wild animals (red deer, sika deer, fallow deer, roe deer, European bison, mouflon, chamois and ibex) and *I. ricinus* ticks. Cluster 5 was restricted to strains from voles and shrews from Europe. Cluster 6 harbored one sample from a blackbird (*T. merula)* and two strains from *I. ricinus* ticks removed from blackbirds. Cluster 7 contained one strain from a horse and two strains from *I. ricinus* ticks. Cluster 8 comprised samples from *I. persulcatus* and *I. pavlovskyi* ticks from the Asian part of Russia as described previously [34]. Cluster 9 was restricted to one sequence [37] from an *Ixodes* sp. tick removed from a woodchat shrike (*Lanius senator senator*). Cluster 10 contained only samples from voles as well as from *I. persulcatus*, *I. pavlovskyi* and their hybrids from the Asian part of Russia as described previously [34]. Clusters 11 and 12 harbored solely strains from North America. Cluster 11 comprised samples from humans, one dog, one horse and small mammals (jumping meadow mouse and chipmunk). Cluster 12 contained strains from humans and *I. scapularis* ticks. One of the tick strains (USG3) has been described as human pathogenic *A. phagocytophilum* Ap-ha strain [38], whereas the other three have been classified as non-human pathogenic *A. phagocytophilum* Ap-variant 1 [36].

Samples from Europe were found in clusters 1–7 and 9, those from Asia in clusters 8 and 10 and those from North America in clusters 11 and 12. Net average distances between the *ankA* gene clusters are shown in Table 4. At the nucleotide level, the highest identity of 97.4% was observed between clusters 1 and 11 and the lowest identity of 47.8% between cluster 1 and cluster 10.

**Table 4:** Net average distances between nucleotide sequences (below the diagonal) and protein sequences (above the diagonal) of *ankA* gene clusters

	Cluster 1	Cluster 2	Cluster 3	Cluster 4	Cluster 5	Cluster 6	Cluster 7	Cluster 8	Cluster 9	Cluster 10	Cluster 11	Cluster 12
Cluster 1	–	73.5	31.1	37.9	41.1	50.7	51.8	86.3	34.3	13.9	95.3	89.7
Cluster 2	80.7	–	42.2	38.3	42.6	52.4	41.9	74.2	38.9	17.4	72.9	74.5
Cluster 3	59.8	71.3	–	11.2	23.5	21.6	8.3	28.2	14.0	2.5	31.5	31.4
Cluster 4	54.0	60.2	49.4	–	64.1	41.7	86.8	33.8	62.4	39.0	34.7	36.4
Cluster 5	59.2	63.4	56.1	81.4	–	42.3	56.9	34.9	58.6	47.9	36.6	36.7
Cluster 6	72.8	73.7	62.4	64.6	67.7	–	31.4	47.7	35.9	21.5	46.7	51.0
Cluster 7	66.7	59.5	48.0	89.3	73.9	56.2	–	39.2	53.8	34.5	47.8	48.8
Cluster 8	93.8	83.5	61.3	56.0	60.1	74.2	63.6	–	27.0	7.9	84.9	84.1
Cluster 9	54.3	61.7	52.5	79.7	81.2	63.2	72.3	54.8	–	42.9	30.7	30.3
Cluster 10	47.8	53.7	50.4	69.6	75.8	59.7	65.3	50.8	71.3	–	13.0	14.4
Cluster 11	97.4	82.3	61.8	54.1	59.1	73.2	65.9	94.2	54.9	48.6	–	91.8
Cluster 12	95.2	83.2	62.7	54.8	58.9	74.2	66.4	94.0	55.4	50.7	97.1	–

Full length *ankA* sequences of the *A. phagocytophilum* strains horse_S1523_07 (present study) and tick_W271 [31] which both belonged to cluster 7 were compared to all other complete *ankA* sequences available so far [30, 31, 39]; the highest identities were observed to cluster 1 (83.9%) and to cluster 4 (86.3%) sequences. However, the identity of cluster 7 sequences was 99.4% to cluster 1 sequences when nucleotides 1–1639 were considered and 98.2% to cluster 4 sequences when nucleotides 1604–3720 were taken into account. This means that cluster 7 *ankA* sequences probably arose by recombination of cluster 1 and cluster 4 sequences.

### Concordance between typing methods

To test for the concordance between different typing methods, adjusted Wallace coefficients [33] were calculated. The information regarding *drhm* presence was obtained in 392 *A. phagocytophilum* strains that were typeable by MLST and *ankA.* The concordance between *drhm* status and continent was 71% (Table 5) which reflects the fact that 94% (478/511) of the European samples, but only 19% (4/21) of those from North America were *drhm*-positive. The association between *drhm* presence and the other partitions was low (host, ST, CC, MLST cluster, *ankA* cluster and country). However, the concordance between ST, CC and *ank*A gene cluster on one hand and *drhm* status on the other hand was high (> 75%), because CC 5, CC 12 and *ankA* gene clusters 11 and 12 were restricted to North American strains (Table 5). The concordance between host and MLST cluster was 88% which indicates a host association of certain *A. phagocytophilum* variants (Table 5). The association between *ankA* cluster and MLST cluster was 96%.

**Table 5:** Adjusted Wallace coefficients and 95% confidence intervals (in parentheses) in percent indicating the concordance between different partitions for the 392 *A. phagocytophilum* strains that were typeable by MLST, *ankA* and *drhm* status

	Host	ST	CC	MLST cluster	*ankA* cluster	*drhm* status	Country of origin	Continent of origin
Host	–	23.0 (18.3–27.6)	61.9 (55.6–68.1)	**88.2** (82.9–93.6)	62.5 (54.1–70.8)	44.9 (25.9–63.9)	37.0 (28.9–45.1)	20.2 (0.0–47.4)
ST	27.8 (23.4–32.3)	–	**100** (100–100)	**100** (100–100)	**99.6** (98.9–100)	**92.5** (82.4–100)	23.9 (14.5–33.3)	**100** (100–100)
CC	13.5 (10.5–16.6)	18.0 (14.7–21.4)	–	**76.2** (70.6–81.7)	**82.0** (79.4–84.6)	**82.8** (68.0–97.6)	8.5 (0.7–16.2)	**96.7** (90.4–100)
MLST cluster	3.2 (1.8–4.7)	3.0 (0.7–5.3)	12.7 (4.8–20.7)	–	30.8 (19.3–42.3)	40.7 (17.4–64.0)	0.0 (0.0–7.4)	1.2 (0.0–43.8)
*ankA* cluster	7.1 (5.0–9.3)	9.4 (6.4–12.3)	42.8 (32.5–53.1)	**96.3** (94.0–98.5)	–	**88.1** (75.7–100)	15.1 (6.1–24.2)	**100** (100–100)
*drhm* status	1.2 (0.0–2.5)	2.0 (0.0–4.2)	9.9 (2.9–16.9)	29.1 (8.7–49.6)	20.2 (8.9–31.4)	–	3.6 (0.0–11.2)	70.9 (49.3–92.5)
Country	7.6 (5.5–9.7)	4.0 (1.8–6.3)	8.0 (0.0–16.2)	0.0 (0.0–27.1)	27.2 (13.1–41.3)	28.5 (0.1–56.9)	–	**100** (100–100)
Continent	0.2 (0.0–1.6)	1.0 (0.0–3.0)	5.4 (0.0–12.1)	0.4 (0.0–23.1)	10.6 (0.0–21.7)	33.0 (9.7–56.2)	5.9 (0.0–13.6)	–

*Note*: Adjusted Wallace coefficients > 75% are marked in bold

The information regarding *drhm* presence was unavailable for all Asian strains. Therefore, adjusted Wallace coefficients were calculated in 467 *A. phagocytophilum* strains that were typeable by MLST and *ankA*, but lacked the partition *drhm* presence. Then, the concordance between MLST cluster and continent was 68% and between *ankA* gene cluster and continent 100% (Table 6).

**Table 6:** Adjusted Wallace coefficients and 95% confidence intervals (in parenthesis) in percent indicating the concordance between different partitions for the 467 *A. phagocytophilum* strains that were typeable by MLST and *ankA*

	Host	ST	CC	MLST cluster	*ankA* cluster	Country of origin	Continent of origin
Host	–	22.3 (18.3–26.4)	62.3 (57.2–67.4)	**83.7** (80.1–87.3)	66.5 (60.2–72.7)	46.2 (39.4–53.0)	**77.1** (69.1–85.2)
ST	29.0 (24.8–33.1)	–	**100** (100–100)	**100** (100–100)	**99.7** (99.2–100)	32.6 (24.5–40.7)	**100** (100–100)
CC	15.6 (12.7–18.6)	19.4 (16.3–22.4)	–	**85.3** (81.9–88.6)	**84.8** (82.8–86.8)	19.3 (12.7–26.0)	**96.9** (93.5–100)
MLST cluster	5.5 (4.0–6.9)	5.0 (2.8–7.2)	22.1 (15.1–29.2)	–	44.2 (34.9–53.5)	9.1 (2.6–15.5)	67.9 (53.9–81.8)
*ankA* cluster	9.6 (7.5–11.7)	11.1 (8.3–13.9)	48.8 (39.7–57.8)	**98.0** (96.7–99.2)	–	23.8 (16.0–31.6)	**100** (100–100)
Country	11.0 (9.0–13.1)	6.0 (3.9–8.1)	18.4 (11.2–25.7)	33.4 (18.2–48.5)	39.4 (28.5–50.4)	–	**100** (100–100)
Continent	2.9 (1.6–4.2)	2.9 (1.0–4.8)	14.7 (8.7–20.6)	39.5 (26.4–52.6)	26.3 (17.3–35.3)	15.9 (9.3–22.5)	–

*Note*: Adjusted Wallace coefficients > 75% are marked in bold

## Discussion

### Presence or absence of the *drhm* gene

The absence of the *drhm* gene in *A. phagocytophilum* strains has been proposed as a marker for pathogenicity in humans and dogs [27]. Eight human strains from North America investigated so far have been *drhm-*negative [27]. Here, we included further human samples from the USA that did not possess *drhm* either. However, all 35 human strains from Europe were *drhm-*positive. Thus, the absence of the *drhm* gene in *A. phagocytophilum* does not seem to be associated with pathogenicity for humans *per se*. On the other hand, *drhm* negativity could indicate that those strains are of higher virulence, because human granulocytic anaplasmosis is infrequently reported from Europe compared to the USA [4].

In contrast to human disease, canine [13] and equine [14] granulocytic anaplasmosis equally occurs in North America and Europe. In a previous study, 25% (4/16) of dog strains and 53% (11/21) of horse strains from the USA were positive for *drhm* [28]. Here, 96% (67/70) of canine and 100% (44/44) of equine samples from Europe possessed the *drhm* gene. Thus, the *drhm* status seems not to be associated with pathogenicity or virulence in dogs and horses. The same is probably true in humans because *A. phagocytophilum* strains from humans, dogs and horses have been reported to be homologous [29, 30, 40] and dogs and horses have been reported to be susceptible to infection with human isolates [41–43].

The concordance between *drhm* status and host and *vice versa* was low. A similar finding has been reported previously [28]. Thus, presence or absence of *drhm* is probably not associated with certain hosts. However, a tendency has been observed for *A. phagocytophilum* strains from the Northeast of the USA to be *drhm-*negative, in contrast to samples from the Southeast, Midwest and West [28]. In the present study, the concordance between *drhm* status and country of origin and *vice versa* was low. Therefore, presence or absence of *drhm* is not geographically informative in Europe. However, 94% (478/511) of the European and 19% (4/21) of the North American *A. phagocytophilum* strains were *drhm-*positive yielding a concordance between *drhm* status and continent of 71%. Thus, *drhm* positivity seems to be associated with European origin, although the concordance was not very strong (< 75%).

Twenty-seven European *A. phagocytophilum* strains were APH_0919/APH_0922- and *drhm-*negative. They originated from voles, shrews and *I. ricinus* ticks removed from blackbirds (*T. merula*) and belonged to MLST clusters 3 and 7. Strains from these clusters were more distantly related to the MLST clusters 1, 2, 4 and 8 for which information on APH_0919/APH_0922 and *drhm* was available. Thus, the most likely reasons for negativity in APH_0919/APH_0922 and *drhm* are primer mismatches or a different genomic organization.

### MLST and *ankA*-based typing

A ST could not be ascribed in 139 *A. phagocytophilum* strains because of double peaks in the chromatograms. This phenomenon has been observed before, most prominently in wild [29] and domestic ruminants [44, 45], probably reflecting their co- or superinfection with different *A. phagocytophilum* variants.

Among others, wild boar and small mammals have been considered in the past as reservoir hosts for human infection in Europe [22]. Here, concatenated housekeeping and *ankA* gene sequences from human strains from Europe clustered most closely together with hedgehogs and wild boar indicating that they might harbor human-pathogenic *A. phagocytophilum* variants. In contrast, samples from voles and shrews from Europe and Asia were only distantly related. Asian *A. phagocytophilum* strains from humans were not available for analysis. Thus, it is unclear whether Asian voles might harbor human-pathogenic variants. At least in Europe, voles and shrews are unlikely to serve as reservoir hosts for human infection.

In the USA, two major *16S* rRNA gene variants of *A. phagocytophilum* have been described: the *A. phagocytophilum* Ap-ha and the *A. phagocytophilum* Ap-variant 1 strain. Both were defined by a two-base pair difference in the *16S* rRNA gene [46] and it has been claimed that *A. phagocytophilum* Ap-ha is pathogenic for humans whereas *A. phagocytophilum* Ap-variant 1 is not [22]. However, single locus *16S* rRNA gene-based typing of *A. phagocytophilum* has been proven to not reliably define *A. phagocytophilum* genotypes [29–31, 47–49]. Here, the *I. scapularis* strains CRT35 (ST 217), CRT38 (ST 216) and CRT53-1 (ST 218) that have been classified as non-human pathogenic *A. phagocytophilum* Ap-variant 1 [36] were found in MLST cluster 8, whereas the human strains from the USA were part of cluster 1. However, concerning the *ankA*-based typing, seven North American strains from humans were part of cluster 12 together with the three *A. phagocytophilum* Ap-variant 1 isolates from *I. scapularis*. In our opinion, this finding questions the classification of *A. phagocytophilum* Ap-variant 1 as non-human pathogenic using the *16S* rRNA gene as a marker.

The epidemiological differences between North America and Europe regarding the incidence of human infection are not explained when MLST and *ankA*-based typing are considered, because human strains from North America and Europe were both part of MLST cluster 1. Further, the North American *ankA* gene clusters 11 and 12 were highly identical at the nucleotide level to the European cluster 1 with 97.4% and 95.2%, respectively.

In contrast to the *drhm* status, the concordance between host and MLST cluster was 88% indicating a host association of certain *A. phagocytophilum* variants. Bird-related MLST cluster 7 and avian *ankA* clusters 6 and 9 are newly described here. Bird-specific *A. phagocytophilum* strains have been reported before because *groEL* ecotype IV was restricted to samples from a blackbird and from five ticks feeding on blackbirds [50]. Recently, a bird-associated *groEL* cluster 7 has been characterized as well [51].

*Ixodes persulcatus*, *I. pavlovskyi* ticks and their hybrids from the Asian part of Russia were restricted to MLST clusters 5 and 6 and *ankA* clusters 8 and 10. *Ixodes persulcatus* from its European distribution area was not investigated. It is therefore unclear whether the clustering is reflected by tick species or geography.

The *ankA* sequences from cluster 7 found in a horse and two *I. ricinus* ticks were probably recombinants of cluster 1 and 4 sequences. It has been shown before that the *ankA* gene might undergo recombination [39]. Here, the infection of a horse with an *A. phagocytophilum* strain of *ankA* cluster 4 was observed for the first time. However, all 44 equine samples of European origin were part of cluster 1. A double or triple infection with *A. phagocytophilum* variants belonging to different *ankA* clusters was detected in 17 cases in cattle, deer and a tick. In roe deer, this phenomenon has been observed before [52]. Thus, multiple infections as a prerequisite for recombination occur.

In general, the genetic diversity was higher in Europe than in North America and Asia because European samples belonged to five MLST and eight *ankA* clusters, whereas North American and Asian strains were part of two MLST and *ankA* clusters. However, a considerable sampling bias must be taken into account, as 585, 72 and 29 strains were of European, Asian and North American origin, respectively.

The concordance between MLST cluster and continent was 68%, and between *ankA* gene cluster and continent 100%. Thus, both typing methods were geographically informative. A broader host spectrum especially from North America and Asia should be typed by MLST and *ankA*-gene-based typing to further elucidate host association and geographical distribution of distinct *A. phagocytophilum* strains.

## Conclusions

The absence of the *drhm* gene in *A. phagocytophilum* does not seem to be associated with pathogenicity for humans *per se*, because all 35 European strains from human granulocytic anaplasmosis cases were *drhm* positive. The epidemiological differences between North America and Europe concerning the incidence of human *A. phagocytophilum* infection are not explained by strain divergence based on MLST and *ankA* gene-based typing. The concordance between host and MLST cluster was 88% which indicates a host association of certain *A. phagocytophilum* strains. Human strains from Europe clustered most closely together with hedgehogs and wild boars indicating that they might serve as reservoir hosts for human infection.

## Supplementary information

**Additional file 1: Table S1**. Reference, host species, ST, CC, MLST cluster, allele numbers, *ankA* gene cluster, *drhm* status, APH_0919/APH_0922 status, country of origin, year of sampling, disease state of the host and GenBank accession numbers for the 686 *A. phagocytophilum* strains.

**Additional file 2: Text S1.** Conditions for amplification by nested PCR and sequencing of the complete open reading frame of *ankA* gene cluster 6.

**Additional file 3: Figure S1.** Phylogenetic tree calculated from the concatenated housekeeping gene sequences of 520 samples without ambiguous nucleotides. Tree construction was achieved by the NJ method using the Jukes-Cantor matrix with the complete deletion option. Bootstrap values ≥ 64% are shown next to the branches. The scale-bar indicates the number of nucleotide substitutions per site. The final data set contained 2877 positions. Identical ST are displayed only once per species. The number in parenthesis indicates the frequency with which the respective ST was found. *Key*: red circles, sequences from humans, dogs, horses and cats; dark blue diamonds, sequences from domestic ruminants; light blue diamonds, sequences from wild ruminants; green triangles, sequences from small mammals; yellow squares, sequences from wild boars; purple triangles, sequences from red foxes; white triangles, sequences from ticks.

**Additional file 4: Figure S2.** Phylogenetic tree calculated from the *ankA* sequences of 623 samples without ambiguous nucleotides. Tree construction was achieved by the NJ method using the Jukes-Cantor matrix with the complete deletion option. Bootstrap values ≥ 69% are shown next to the branches. The scale-bar indicates the number of nucleotide substitutions per site. The final data set contained 510 positions. Identical *ankA* sequences are displayed only once per species. The number in parenthesis indicates the frequency with which the respective sequence was found. *Key*: red circles, sequences from humans, dogs, horses and cats; dark blue diamonds, sequences from domestic ruminants; light blue diamonds, sequences from wild ruminants; green triangles, sequences from small mammals; yellow squares, sequences from wild boars; purple triangles, sequences from red foxes; pink square, sequence from a bird, white triangles, sequences from ticks.

## Data Availability

All data generated or analysed during this study are included in this published article and its additional files. All nucleotide sequences are available at GenBank. GenBank accession numbers are shown in Additional file 1: Table S1. The MLST profiles for samples without ambiguous nucleotides were submitted to the *A. phagocytophilum* isolates database hosted on PubMLST (https://pubmlst.org/aphagocytophilum/).

## Abbreviations

CC: Clonal complex
MLST: Multilocus sequence typing
NJ: Neighbor-joining
SNP: Single nucleotide polymorphism
ST: Sequence type
