# Correction to: The absence of the *drhm* gene is not a marker for human-pathogenicity in European *Anaplasma phagocytophilum* strains

**DOI:** 10.1186/s13071-020-04350-5

**Published:** 2020-09-30

**Authors:** Denis B. Langenwalder, Sabine Schmidt, Cornelia Silaghi, Jasmin Skuballa, Nikola Pantchev, Ioana A. Matei, Andrei D. Mihalca, Urs Gilli, Joanna Zajkowska, Martin Ganter, Tove Hoffman, Erik Salaneck, Miroslav Petrovec, Friederike D. von Loewenich

**Affiliations:** 1 Department of Medical Microbiology and Hygiene, Medical Center of the Johannes Gutenberg-University Mainz, Obere Zahlbacherstrasse 67, 55131 Mainz, Germany; 2 Institute of Infectology, Friedrich-Loeffler-Institut, Südufer 10, 17493 Greifswald - Insel Riems, Germany; 3 Chemical and Veterinary Investigations Office Karlsruhe (CVUA Karlsruhe), Weissenburgerstrasse 3, 76187 Karlsruhe, Germany; 4 IDEXX Laboratories, Mörikestrasse 28/3, 71636 Ludwigsburg, Germany; 5 Department of Parasitology and Parasitic Diseases, University of Agricultural Sciences and Veterinary Medicine of Cluj-Napoca, Calea Manastur 3-5, Cluj-Napoca, 400372 Romania; 6 IDEXX Diavet AG, Schlyffistrasse 10, 8806 Bäch, Switzerland; 7 Department of Infectious Diseases and Neuroinfections, Medical University of Białystok, ul.Żurawia 14, Białystok, 15-345 Poland; 8 Clinic for Swine and Small Ruminants, University of Veterinary Medicine Hannover, Bischofsholer Damm 15, 30173 Hannover, Germany; 9 Department of Medical Biochemistry and Microbiology (IMBIM), Zoonosis Science Center, Uppsala University, Uppsala, Sweden; 10 Department of Medical Sciences, Zoonosis Science Center, Uppsala University, Uppsala, Sweden; 11 Miroslav Petrovec, Institute of Microbiology and Immunology, Faculty of Medicine, University of Ljubljana, Zaloška 4, 1000 Ljubljana, Slovenia

## Correction to: Parasites Vectors (2020) 13:238 10.1186/s13071-020-04116-z

Following publication of the original article [1], the author flagged that unfortunately there are errors in some of the figures and additional files.

The color-coding in Figs. 1 and 2, and in Additional file 3: Figure S1 and Additional file 4: Figure S2 is wrong: Bison samples are displayed in dark blue instead of light blue.

In addition, some numbers for the *atpA* alleles in Additional file 1: Table S1 are wrong.

The corrected versions of Figs. 1 and 2, and Additional files 1, 3 and 4 are provided in this correction.

The authors apologize for the inconvenience caused.

**Fig. 1:**
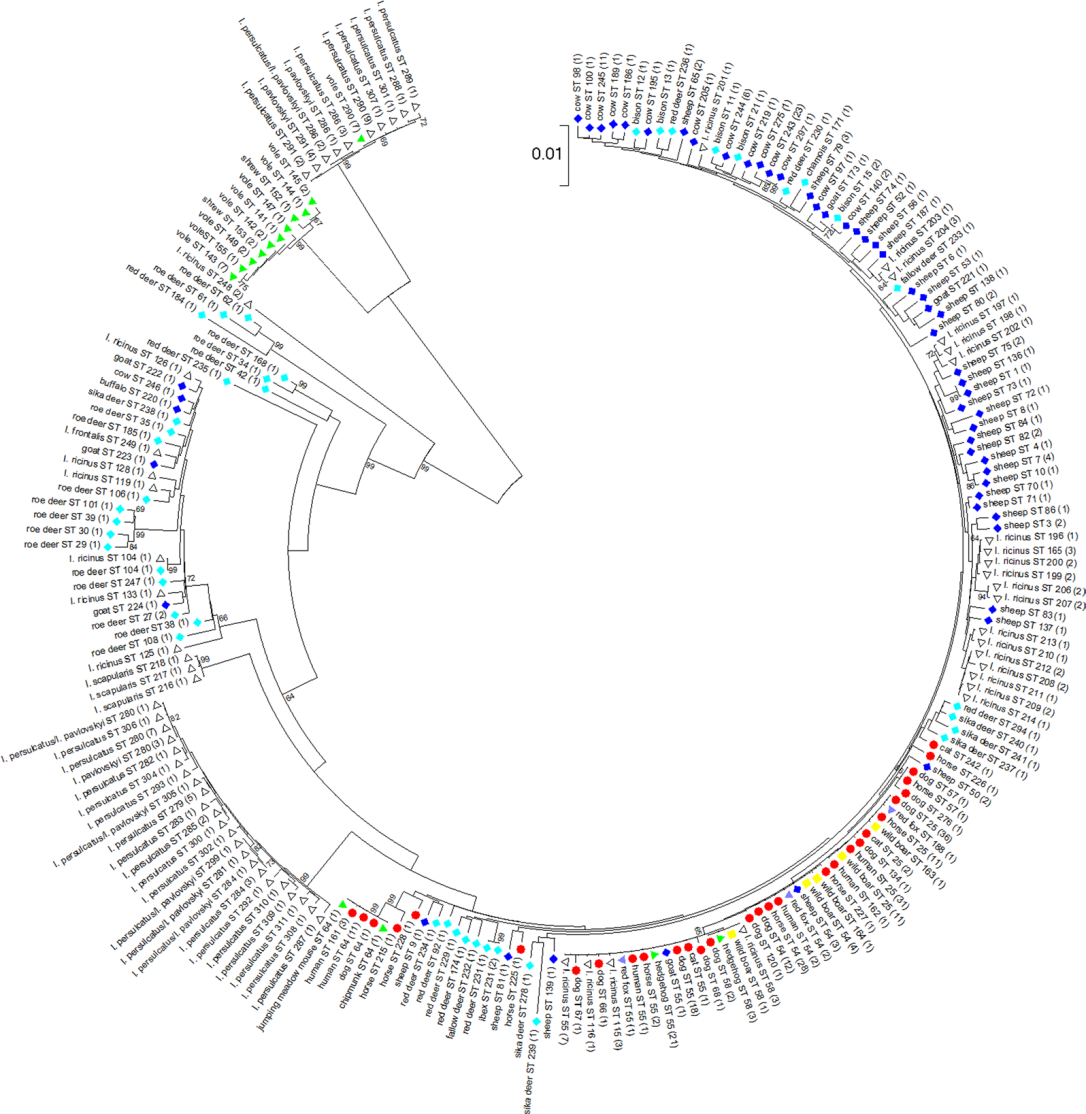
Phylogenetic tree calculated from the concatenated housekeeping gene sequences of 520 samples without ambiguous nucleotides. Tree construction was achieved by the NJ method using the Jukes-Cantor matrix with the complete deletion option. Bootstrap values ≥ 64% are shown next to the branches. The scale-bar indicates the number of nucleotide substitutions per site. The final data set contained 2877 positions. Identical ST are displayed only once per species. The number in parenthesis indicates the frequency with which the respective ST was found. *Key*: red circles, sequences from humans, dogs, horses and cats; dark blue diamonds, sequences from domestic ruminants (cattle, sheep, goats and water buffalo); light blue diamonds, sequences from wild ruminants (roe deer, red deer, sika deer, fallow deer, European bison, mouflon, chamois and ibex); green triangles, sequences from small mammals (hedgehogs, voles, shrews, chipmunk and jumping meadow mouse); yellow squares, sequences from wild boars; purple triangles, sequences from red foxes; white triangles, sequences from ticks

**Fig. 2:**
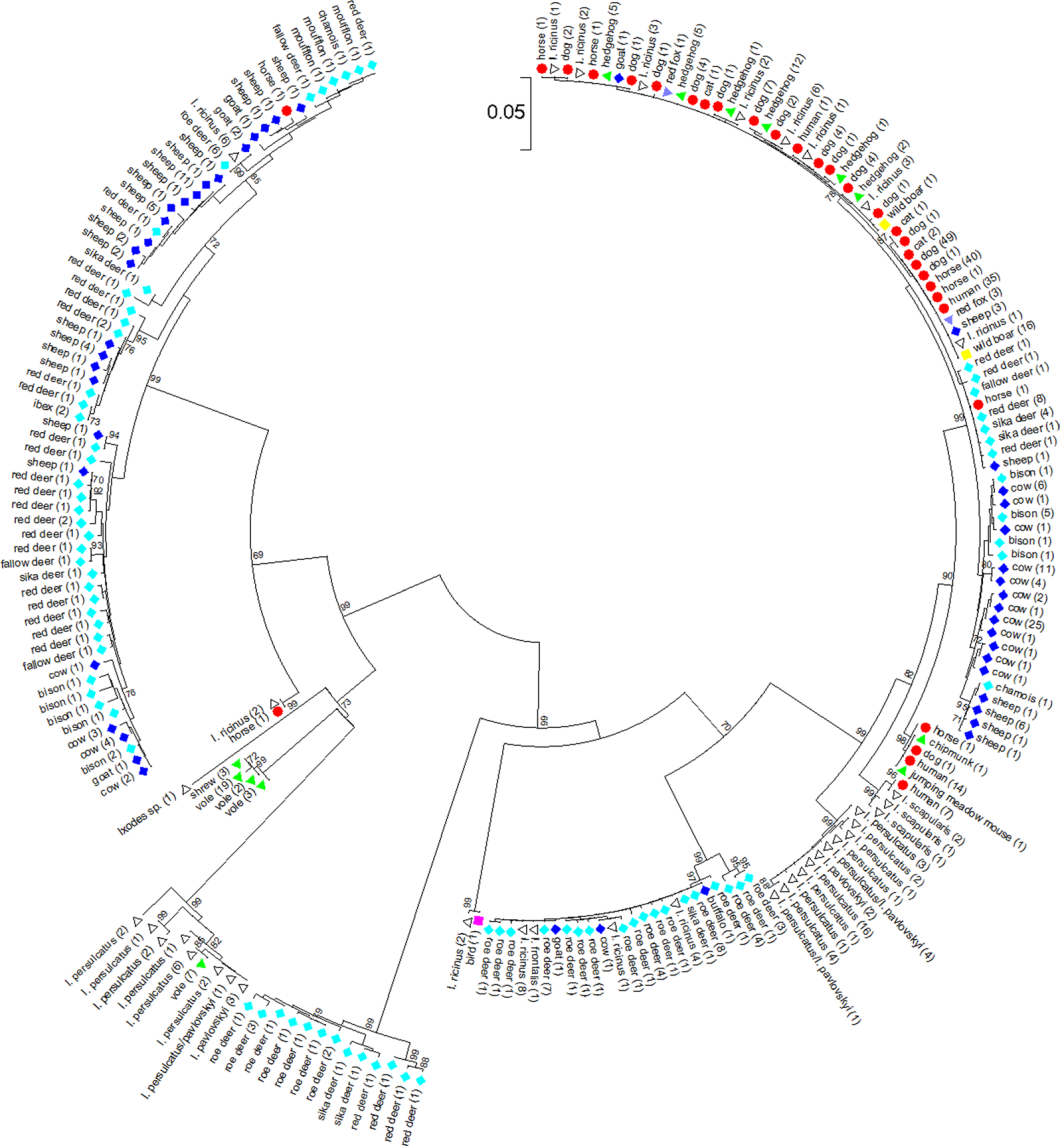
Phylogenetic tree calculated from the *ankA* sequences of 623 samples without ambiguous nucleotides. Tree construction was achieved by the NJ method using the Jukes-Cantor matrix with the complete deletion option. Bootstrap values ≥ 69% are shown next to the branches. The scale-bar indicates the number of nucleotide substitutions per site. The final data set contained 510 positions. Identical *ankA* sequences are displayed only once per species. The number in parenthesis indicates the frequency with which the respective sequence was found. *Key*: red circles, sequences from humans, dogs, horses and cats; dark blue diamonds, sequences from domestic ruminants (cattle, sheep, goats and water buffalo); light blue diamonds, sequences from wild ruminants (roe deer, red deer, sika deer, fallow deer, European bison, mouflon, chamois and ibex); green triangles, sequences from small mammals (hedgehogs, voles, shrews, chipmunk and jumping meadow mouse); yellow squares, sequences from wild boars; purple triangles, sequences from red foxes; pink square, sequence from a bird, white triangles, sequences from ticks

## Supplementary information

**Additional file 1: Table S1**. Reference, host species, ST, CC, MLST cluster, allele numbers, *ankA* gene cluster, *drhm* status, APH_0919/APH_0922 status, country of origin, year of sampling, disease state of the host and GenBank accession numbers for the 686 *A. phagocytophilum* strains.

**Additional file 3: Figure S1.** Phylogenetic tree calculated from the concatenated housekeeping gene sequences of 520 samples without ambiguous nucleotides. Tree construction was achieved by the NJ method using the Jukes-Cantor matrix with the complete deletion option. Bootstrap values ≥ 64% are shown next to the branches. The scale-bar indicates the number of nucleotide substitutions per site. The final data set contained 2877 positions. Identical ST are displayed only once per species. The number in parenthesis indicates the frequency with which the respective ST was found. *Key*: red circles, sequences from humans, dogs, horses and cats; dark blue diamonds, sequences from domestic ruminants; light blue diamonds, sequences from wild ruminants; green triangles, sequences from small mammals; yellow squares, sequences from wild boars; purple triangles, sequences from red foxes; white triangles, sequences from ticks.

**Additional file 4: Figure S2.** Phylogenetic tree calculated from the *ankA* sequences of 623 samples without ambiguous nucleotides. Tree construction was achieved by the NJ method using the Jukes-Cantor matrix with the complete deletion option. Bootstrap values ≥ 69% are shown next to the branches. The scale-bar indicates the number of nucleotide substitutions per site. The final data set contained 510 positions. Identical *ankA* sequences are displayed only once per species. The number in parenthesis indicates the frequency with which the respective sequence was found. *Key*: red circles, sequences from humans, dogs, horses and cats; dark blue diamonds, sequences from domestic ruminants; light blue diamonds, sequences from wild ruminants; green triangles, sequences from small mammals; yellow squares, sequences from wild boars; purple triangles, sequences from red foxes; pink square, sequence from a bird, white triangles, sequences from ticks.
