# Supplementary material for: The absence of the drhm gene is not a marker for human-pathogenicity in European Anaplasma phagocytophilum strains
Source: Parasit Vectors. 2020 May 7;13:238. doi: 10.1186/s13071-020-04116-z (PMC7206706; doi:10.1186/s13071-020-04116-z)
Supplement: Supplementary file 2 — Additional file 2: Text S1. Conditions for amplification by nested PCR and sequencing of the complete open reading frame of ankA gene cluster 6. [file 13071_2020_4116_MOESM2_ESM.docx]

**Additional file 2: Text S1** Conditions for amplification by nested PCR and sequencing of the complete open reading frame of *ankA* gene cluster 6

**Reaction mixture for amplification:**

Reagent Volume Concentration

Buffer 10 x 5.0 µl 1 x

dNTP 2 mM 5.0 µl 200 µM

MgCl_2_ 50 mM 2.0 µl 2 mM

Primer 1 20 µM 1.0 µl 400 nM

Primer 2 20 µM 1.0 µl 400 nM

*Taq* DNA polymerase 0.2 µl 1 U

DNA 2.0 µl

Water 33.8 µl

**PCR conditions for amplification:**

Initial denaturation 94°C 3 min

Denaturation 94°C 30 s

Annealing see below 30 s

Extension 72°C see below

Finale Extension 72°C 10 min

Cycles 40

| First round | Annealing | Extension | Second round | Annealing | Extension |
| --- | --- | --- | --- | --- | --- |
| Nager U8  Amsel beg re1 | 50°C | 2 min | Nager U8  Amsel beg re2 | 50°C | 2 min |
| SLO fo1  SLO re1 | 52°C | 1 min | SLO fo1  Amsel beg re1 | 52°C | 30 s |
| SLO fo1  SLO re1 | 52°C | 1 min | SLO fo2  SLO re2 | 52°C | 30 s |
| Amsel mi fo1  D5 | 52°C | 2 min | Amsel mi fo2  D5 | 52°C | 2 min |

**Sequencing:**

In general, the same primers as for amplification were used for bidirectional sequencing. The PCR product of primers Amsel mi fo2 and D5 was additionally sequenced with the following primers:

Amsel mi seq fo1

Amsel mi seq fo2

Amsel mi seq re2

**Primer sequences (5’ – 3’):**

Amsel beg re1: GAA GTT TCC ATC CTT TGC TGC

Amsel beg re2: AAT GTT CCT GCA TCT TGT GTA

Amsel mi fo1: CAA ATA CAA CTA TTA CCG CCG A

Amsel mi fo2: TGA AAG TGT TAA TTA AAG CAG GT

Amsel mi seq fo1: ATA CGT CAT TGC ATA GTA GTC

Amsel mi seq fo2: ATA CAT GTG TAT TGG CTG ATG

Amsel mi seq re2: CTG TTT CTT CAG GTG GCA CA

D5: GTA YCT CAG CGA TTT TKC GGT AT

Nager U8: ATT GAA TAA AGG CCC CAA CA

SLO fo1: GGG ATR AGT GCR GTG CAG YAT

SLO fo2: TTA CGC TGT RRT RGC ATR GAC

SLO re1: ACT GCR GCM GCT ARA GGR CT

SLO re2: AWR GWT CCS KYA GGA GYA TTT A
