# Supplementary figures and images for: The absence of the drhm gene is not a marker for human-pathogenicity in European Anaplasma phagocytophilum strains
Source: Parasit Vectors. 2020 May 7;13:238. doi: 10.1186/s13071-020-04116-z (PMC7206706; doi:10.1186/s13071-020-04116-z)

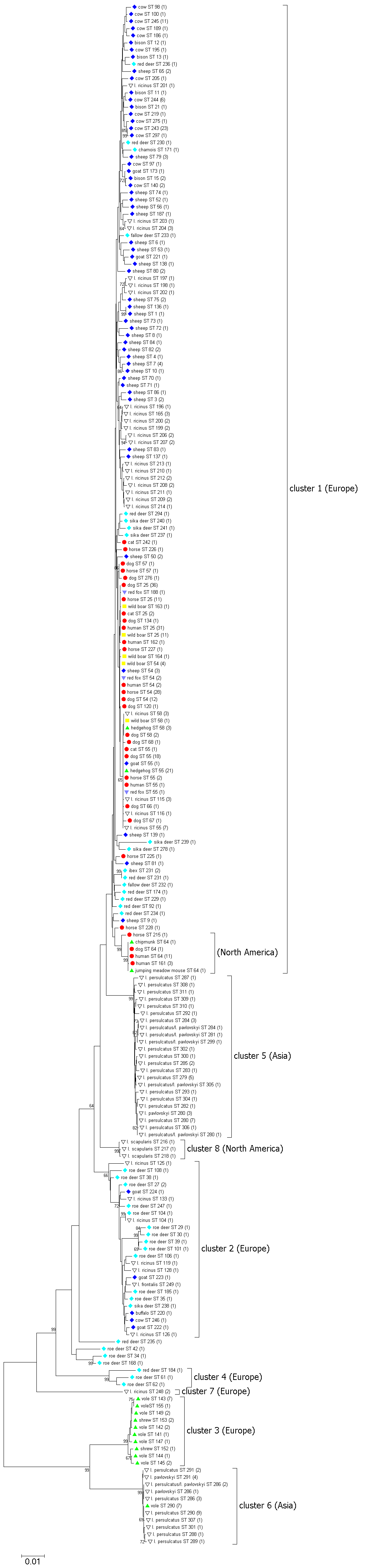

Supplement: Supplementary file 3 — Additional file 3: Figure S1. Phylogenetic tree calculated from the concatenated housekeeping gene sequences of 520 samples without ambiguous nucleotides. Tree construction was achieved by the NJ method using the Jukes-Cantor matrix with the complete deletion option. Bootstrap values ≥ 64% are shown next to the branches. The scale-bar indicates the number of nucleotide substitutions per site. The final data set contained 2877 positions. Identical ST are displayed only once per species. The number in parenthesis indicates the frequency with which the respective ST was found. Key: red circles, sequences from humans, dogs, horses and cats; dark blue diamonds, sequences from domestic ruminants; light blue diamonds, sequences from wild ruminants; green triangles, sequences from small mammals; yellow squares, sequences from wild boars; purple triangles, sequences from red foxes; white triangles, sequences from ticks. [file 13071_2020_4116_MOESM3_ESM.tif]

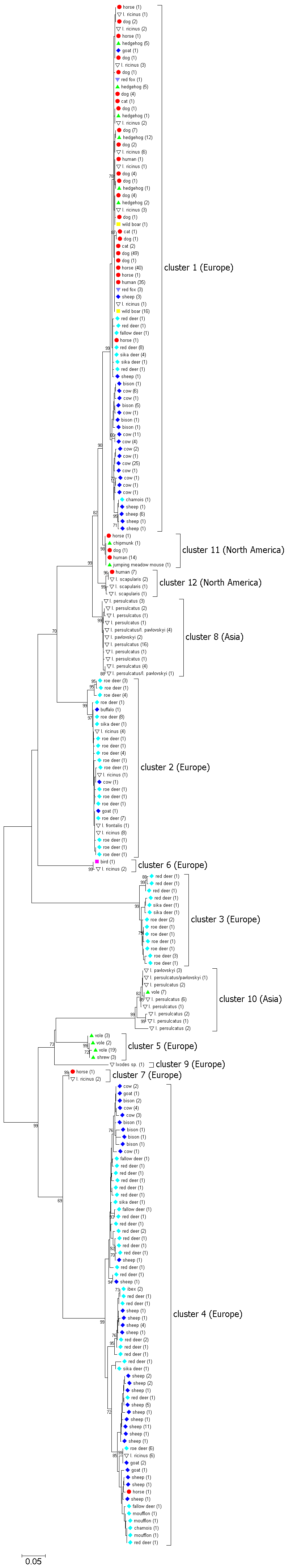

Supplement: Supplementary file 4 — Additional file 4: Figure S2. Phylogenetic tree calculated from the ankA sequences of 623 samples without ambiguous nucleotides. Tree construction was achieved by the NJ method using the Jukes-Cantor matrix with the complete deletion option. Bootstrap values ≥ 69% are shown next to the branches. The scale-bar indicates the number of nucleotide substitutions per site. The final data set contained 510 positions. Identical ankA sequences are displayed only once per species. The number in parenthesis indicates the frequency with which the respective sequence was found. Key: red circles, sequences from humans, dogs, horses and cats; dark blue diamonds, sequences from domestic ruminants; light blue diamonds, sequences from wild ruminants; green triangles, sequences from small mammals; yellow squares, sequences from wild boars; purple triangles, sequences from red foxes; pink square, sequence from a bird, white triangles, sequences from ticks. [file 13071_2020_4116_MOESM4_ESM.tif]
